# Analysis of Chemical Oxygen Demand in Barrel Finishing Based on Reusing Water Resource of Grinding Fluid

**DOI:** 10.3390/ma17164051

**Published:** 2024-08-15

**Authors:** Huiting Shi, Xuenan Li, Shengqiang Yang, Ruihao Zhao, Xiang Yuan

**Affiliations:** 1 School of Mechanical Engineering, Taiyuan University of Science and Technology, Taiyuan 030024, China; 2022032@tyust.edu.cn (R.Z.); 2022033@tyust.edu.cn (X.Y.); 2 College of Mechanical and Vehicle Engineering, Taiyuan University of Technology, Taiyuan 030024, China; xuenan_li@foxmail.com (X.L.); tutysq@263.net.cn (S.Y.); 3 Shanxi Province Key Laboratory of Precise Machining, Taiyuan 030024, China

**Keywords:** grinding fluid, barrel finishing, GO, COD, water quality index

## Abstract

To explore the sustainable development of grinding fluid in barrel finishing, the idea of water resource reuse in grinding fluid has been proposed. The influence of the graphene oxide (GO) and the sodium dodecyl benzene sulfonate (SDBS) as main components in the grinding fluid on the chemical oxygen demand (COD) was analyzed. Repreparing new grinding fluids by utilizing the water resources in grinding fluid after finishing will not cause a sharp increase in COD value. GO which absorbs SDBS can be taken away from grinding fluid by physical separation. It will decrease the COD value of grinding fluid. However, SDBS exists in the form of colloids in the grinding fluid and cannot be removed through physical separation, which also affects the COD value. Based on water quality indicators (the COD, pH, total hardness, metal aluminum, anionic surfactants, and total dissolved solids), the water quality index (WQI) of the reusing grinding fluid after finishing by the physical separation is significantly reduced. It indicates that reusing water resources in grinding fluid is a feasible way to reuse grinding fluid.

## 1. Introduction

Barrel finishing is a micro grinding process, which is realized by a forced flow field composed of a liquid substance (grinding fluid) and solid particle (block shaped media). Compared to shot peening, it has smaller contact force, which can achieve better surface roughness [1]. Compared to burnishing, it is more suitable for batch processing of complex structural parts because of the use of free abrasive tool In addition, barrel finishing has the characteristics of high economic efficiency and simple operation. At present, barrel finishing has been widely applied in the fields of biomedical sciences [2], automotive manufacturing [3], and aerospace [4,5].

Grinding fluid is defined as a kind of liquid that can remarkably lubricate, clean, and soften the surface of parts. Typically, the roles of grinding fluid in barrel finishing are improving processing efficiency and improving the surface quality of parts. Its ingredients are commonly water, an oiliness agent, surfactant, and additives. Oiliness agents act as a lubricant which makes the surfaces of the parts smoother [6]. Surfactant maintains the stabilities of grinding fluid [7,8,9], and its wetting effect can ensure the parts roughness and cleanness [10]. As for additives, they serve as a functional component with their improvement of corrosion resistance, defoaming, and disinfecting.

Industrial wastewater is inevitably generated due to the use of grinding fluid in finishing. The chemical oxygen demand (COD) value was widely used to evaluate the wastewater discharge [11]. The higher the COD value is, the more serious the water pollution is [12,13]. The COD value exceeded 20,000 when traditional grinding fluid was used in barrel finishing. It significantly increases environmental problems. In the face of such problems, many companies or researchers are concerned about wastewater post-treatment processes. For example, anaerobic digestion [14] is a biological process, and electro-oxidation [15] is electrochemical process. They are commonly used for removing the COD of wastewater. In addition, the efficiency of pollutant degradation has been improved by combining biological and electrochemical processes for wastewater treatment. However, the control and operation of biological processes are complex, and the economic benefits of electrochemical processes are poor. The direct method to solve the problem of high chemical oxygen demand in grinding fluid is to develop new kinds of grinding fluid. However, the formulas for grinding fluids are rarely disclosed due to market competition. The influence mechanism of grinding fluid on COD is also rarely studied. Lack of guidance from existing grinding fluid formulas and the influence mechanism of grinding fluid on COD hinders the development of new grinding fluid.

Recently, Minimal Quantity Lubrication (MQL) as a near dry processing technology has been able to minimize the use of liquid medium and reduce the pressure of wastewater treatment in the later stage. MQL is widely used in grinding and other processes, becoming a choice for achieving clean production [16,17,18]. Nanoparticles are the main component of MQL technology. It can form a protective film at the contact interface to enhance lubrication and effectively reduce the friction coefficient at the contact interface. Hard nanoparticles can also polish high points on the surface to reduce surface roughness and reduce friction [19,20]. This indicates that nanoparticles can be applied to grinding fluids for barrel finishing. In addition, nanoparticles such as graphene oxide (GO) have good hydrophilicity and high specific surface area. Their large adsorption capacity enables them to remove metal cations and organic complexes from water, making them an effective adsorbent for the wastewater treatment processes [21]. And, it has been known that the result of multiple mechanisms (such as surface adsorption, chemisorption, coagulation, and precipitation) acting simultaneously can remove COD [22,23]. For example, fly ash is used as adsorbent in water treatment, which can be identified as a good way to sustainably use resources [24]. Although, Graphene oxide (GO), with low toxicity, affects the environment and animal health [25]. However, Ghulam [26] points out that one can apply graphene-based nanomaterials under safe conditions (size, dosage, exposure time, and the functionalized compounds). In our previous study, graphene oxide (GO), in place of an oiliness agent, was introduced into the grinding fluid as the lubricant additive [27]. However, we have not studied the effect of GO adsorption on COD removal.

Therefore, physical separation of the solid medium in grinding fluid and reusing liquid medium in grinding fluid were considered based on the grinding fluid with GO. Through experiment and characterization, the influence mechanism of grinding fluid on COD was studied. This could guide the development of new grinding fluids with low COD values, which would be beneficial for environmental protection. Besides COD, the pH, total hardness, metal aluminum, anionic surfactants, and total dissolved solids as water quality parameters were characterized. Based on these water quality parameters, the water quality of reused grinding fluid was evaluated. And, the finishing effect of the reused grinding fluid was researched by experiment. It shows that reusing liquid medium in grinding fluid can be a suitable method for green development of barrel finishing.

## 2. Materials and Methods

### 2.1. Liquid Medium

**Figure 1:**
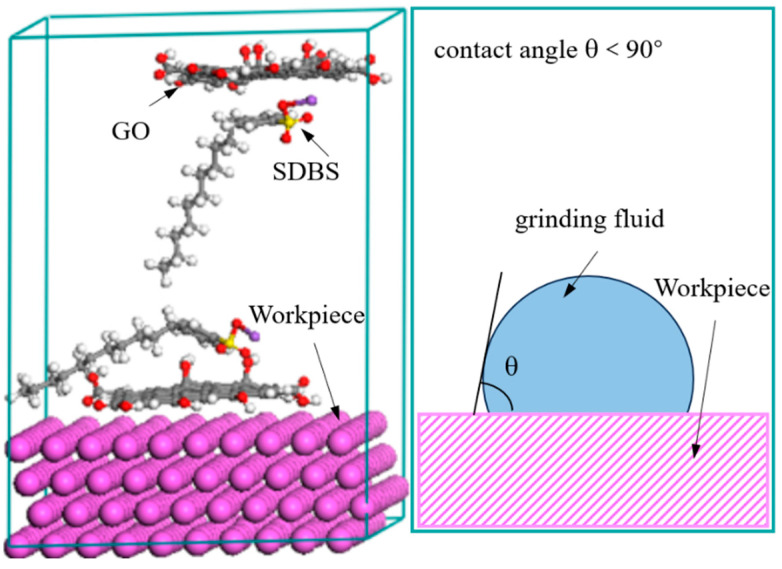
Contact characteristics between grinding fluid and workpiece.

The composition of the grinding fluid for barrel finishing is deionized water (400 mL), GO (0.2 g), and the sodium dodecyl benzene sulfonate (SDBS, 0.4 g). The thickness of the GO is 1 nm, and the particle size of the GO is about 5~100 μm. For preparing grinding fluid, the GO was added into deionized water and stirred for 60 min by ultrasonic method at room temperature. Then, the sodium dodecyl benzene sulfonate (SDBS) was added into the solution. The SDBS adsorbed on GO based on self-assembly properties, by the Coulomb interaction and the van der Waals interaction, and the contact angle between the grinding fluid and workpiece is less than 90°, as shown in Figure 1 [27]. The self-assembly of molecules represents that the GO can be stably dispersed in the grinding fluid. It shows that the grinding fluid has a certain degree of stability. The grinding fluid and workpiece will always maintain microscopic contact when the contact angle is less than 90° between them. This means that the grinding fluid has a certain degree of wettability. That is to say that the grinding fluid can continuously work on the surface of the workpiece, making the surface roughness of workpiece better.

The method of reusing water resources in grinding fluid is referred to as physical separation filtration. The separation process is first accelerated using a centrifugal separator (800-1, Taobao, Changzhou, China), and then filtered and separated using filter paper with a pore size of 1–3 μm. Ten different liquid mediums are discussed in the paper. Solution A1 is the grinding fluid for barrel finishing. For comparison to solution A1, the other nine liquid mediums are discussed. Solution A2 is the liquid medium from solution A1 after barrel finishing for 50 min. The solution A3 is the reused water resources filtered from solution A2. GO and SDBS are added to solution A3 to form a new solution, A4. The solution A5 is the liquid medium from the solution A4 after barrel finishing for 50 min. The solution A6 is the reused water resources filtered from solution A5. Solution B1 is the liquid medium composed deionized water and GO. Solution B2 is the liquid medium from solution B1 after barrel finishing for 50 min. Solution C1 is the liquid medium composed of deionized water and GO. Solution C2 is the liquid medium from solution C1 after barrel finishing 50 min. The specific information on the liquid medium is shown in Table 1. Ten liquid mediums were characterized for COD. Solutions (A1, A5, A6) were characterized by the pH, total hardness, metal aluminum content, anionic surfactants, and total dissolved solids.

### 2.2. Characterizations

The COD, pH, total hardness, metal aluminum, anionic surfactants, and total dissolved solids were evaluating indicators for water and wastewater [28]. COD is measured by the dichromate method, and the detection equipment is the standard COD digestion instrument (HCA-102, Nanjing Huanke Analytical Instrument Corporation, Nanjing, China). Using the pH tester (PH818, Shenzhen Jige Electromechanical Equipment Corporation, Shenzhen, China), the pH value of grinding fluid is obtained. By electric blast drying oven (DHG-9140A, Shanghai Kexiao Scientific Instrument Corporation, Shanghai, China), the total amount of calcium and magnesium are determined, using EDTA titration to determine the total hardness of the grinding fluid. Using inductively coupled plasma emission spectrometer (ICAPPRO, Shanghai Zhuangrun International Trade Corporation, Shanghai, China) the content of aluminum metal is determined. The ultraviolet visible spectrophotometer (T6, Tianjin Telusi Corporation, Tianjin, China) is used to determine anionic surfactants based on the methylene blue spectrophotometric method. Using electronic balance (SQP, Sartorius, Berlin, Germany), the amount of total dissolved solids is determined. Using metallurgical microscope (NX30T-3M180, AOSVI, Ningbo, China), the grinding fluid was photographed.

### 2.3. Barrel Finishing

**Figure 2:**
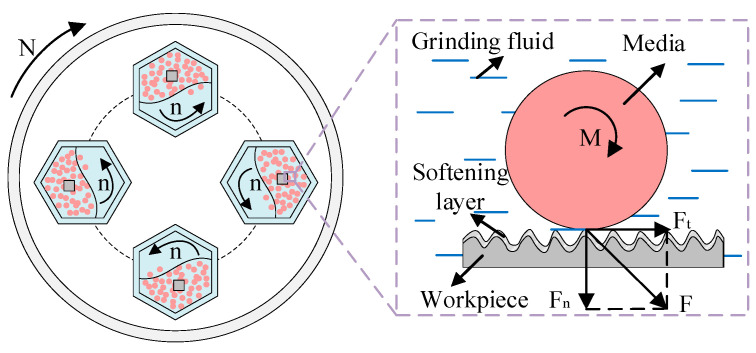
Principal diagram of barrel finishing.

For the barrel finishing experiment, the vertical centrifugal barrel finishing equipment (BJL-LL05, Northern Tianyu Electromechanical Technology Corporation, Langfang, China) is selected. As shown in Figure 2, the workpiece, media, and grinding fluid (solution A1) were loaded into four sealed drums in a certain proportion. The equipment adopts planetary motion. The base rotates, and the four drums rotate in the opposite direction by autorotation at the same rate. It causes collision, rolling, and sliding effects on the surface of the workpiece by media. This leads to a cutting or grinding operation which produces good surface roughness [29], and grinding fluid softening of the workpiece surface at the contact interface. The rotation speed (N) of finishing equipment is 300 r/min, and the opposite direction rotation (n) of drums is −300 r/min. The solid finishing medium is white ceramic media with a diameter of 4 mm. The main composition of the media is Al_2_O_3_, and its particle strength (the radial compressive strength) exceeds 3500 N·cm^−1^. The workpiece is a 7075-aluminum (GB/T) alloy with dimensions of 20 mm × 20 mm × 5 mm. Its hardness is 150 HB, and its main alloying elements are Mg and Zn.

## 3. Results and Discussion

### 3.1. The COD of Grinding Fluid

**Figure 3:**
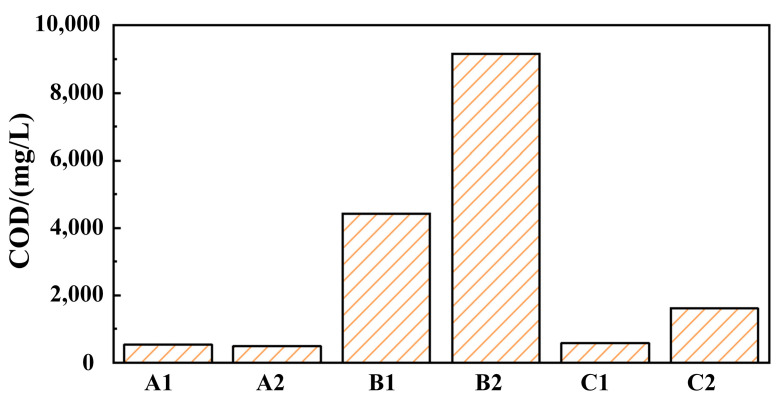
The COD values of different solutions (A1, A2, B1, B2, C1, C2).

The COD values of grinding fluid, GO solution, and SDBS solution before and after finishing were obtained, as shown in Figure 3. The COD values of grinding fluid before finishing (solution A1) and after finishing (solution A2) are 526 and 486, respectively, which indicates that the COD value of the grinding fluid is significantly lower than that of the traditional grinding fluid. And, the COD value of the grinding fluid can be greatly reduced using nanoparticles instead of an oiliness agent, which provides a feasible direction for the green development of grinding fluid in barrel finishing. The COD values of GO solution before finishing (solution B1), GO solution after finishing (solution B2), SDBS solution before finishing (solution C1), and SDBS solution before finishing (solution C2) are higher than those of A1 and A2. This indicates that the self-assembly of GO and SDBS leads to forming new molecules with new structures and properties, reducing the COD value of grinding fluid.

**Figure 4:**
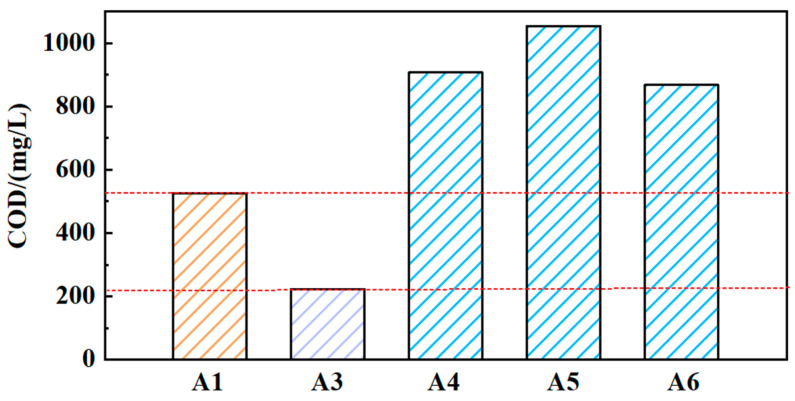
The COD values of different solutions (A1, A3, A4, A5, A6).

The COD values of solutions (A3, A4, A5, and A6) were obtained, as shown in Figure 4. Compared to solution A1, the COD value of solution A3 has decreased to 223. It indicates that the solid medium GO can affect the COD value of the grinding fluid. The COD values of solutions A4, A5, and A6 are all higher than those of solutions A1 and A2. This indicates that the reused grinding fluid will accumulate COD values. However, the COD values of solutions A4, A5, and A6 have not increased dramatically. Although there are still substances that affect the COD values in the grinding fluid containing nanoparticles during the reusing process, a small increase in COD will not affect the reuse of grinding fluid.

**Figure 5:**
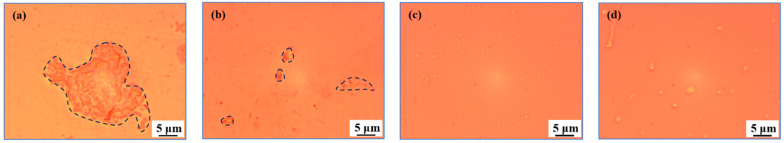
The micrograph of solutions: (**a**) A1; (**b**) A2; (**c**) A3; (**d**) A6.

As shown in Figure 5a, it is found that the solution A1 has irregular flaky solid substances with sizes greater than 5 μm through a metallurgical microscope. The solid substances were considered as GO; that is, the main component of grinding fluid. Observing the solution A2, smaller solid substances were uniformly dispersed in it, as shown in Figure 5b. These smaller solid substances may be small grinding debris separated from the surface of media or workpiece during barrel finishing. It may also be formed by the crushing and separation of GO during barrel finishing, as shown in Figure 5a. In addition, there are still obvious single-layer flakes of GO in solution A2 after barrel finishing, as shown in Figure 5b. This indicates that the 50 min barrel finishing process for did not completely destroy the GO and the grinding fluid still has a certain processing performance.

Comparative to what was observed of c, the COD value of solution A3 is lower than that of solution A1 and the COD value of the grinding fluid A6 is lower than that of solution A5. In addition, both solution A3 and solution A6 after separation and filtration have changed from a black state to a colorless state. It shows that the GO and impurities generated during finishing are almost completely removed, and there are still non-solid substances that affect COD. Through optical microscope observation, it is found that there are still granular colloidal substances in solutions A3 and A6, as shown in Figure 5c,d. And, the size of the granular colloidal substances in solution A6 is slightly larger than that in solution A3. GO in the grinding fluid can be used as an adsorbent to remove a certain amount of surfactant SDBS. However, a portion of SDBS will still remain in the liquid when SDBS is excessive. SDBS as surfactant will aggregate to form a certain amount of colloid in the liquid, affecting the COD value.

### 3.2. Water Quality Analysis of Grinding Fluid

In order to explore the discharge issue of grinding fluid, the pH, total hardness, metal aluminum content, anionic surfactants, and total dissolved solids are analyzed. Solution A1, A5, and A6 were tested, and the results are shown in Table 2.

Grinding fluid may cause microbial contamination during long-term use. The growth of these bacteria and fungi causes the grinding fluid to become rancid, significantly shortening the lifespan of the grinding fluid [30]. When the pH value is high, it can slow down the production of fungi and reduce alloy corrosion. But, excessive pH value can cause human health issues, such as skin pain [31]. Therefore, the pH value of grinding fluid is best when maintained within a range of 8.6 to 9.3. As shown in Table 1, the pH values of the three grinding fluids are all between 6.5 and 8.5, meeting the conventional water quality standards. Compared to solution A1, the pH value of solution A3 is lower. This indicates that there is a certain chemical effect in the barrel finishing which can cause the grinding fluid to become acidic. In addition, the pH value of solution A5 is higher than that of solution A3. This shows that reusing grinding fluid does not increase the risk of microbial contamination or guarantee the service life of the grinding fluid.

The total hardness of water refers to the total concentration of calcium and magnesium ions contained in the water. When the hardness of water is too high, it can affect the finishing ability. For example, thick scale could be produced, affecting shape accuracy, when large-sized or complexly shaped parts were being finished [32]. Therefore, the total hardness of the liquid medium during the finishing also needs to be carefully considered. From Table 2, it can be seen that the total hardness values of the three grinding fluids all meet the conventional water quality standards (less than 450 mg/L) and belong to soft water. On the one hand, GO solution as soft water can effectively inhibit bacterial growth. The grinding fluid containing nanoparticles with soft water properties is beneficial for delaying the acidification phenomenon, ensuring service life. On the other hand, grinding fluids with soft water properties reduce the probability of combining calcium and magnesium ions with SDBS to form surfactant precipitates, ensuring its chemical activity.

The media used in barrel finishing are made of alumina abrasive and are binder sintered [33]. Additionally, the workpieces used are aluminum alloy parts, which inevitably leads to an increase in aluminum content in the grinding fluid. Aluminum mainly exists in aquatic ecosystems in the form of inorganic monomer aluminum (Al^3+^, AlOH^2+^, Al(OH)_2_^+^), amorphous flocs (Al(OH)_3_), and polymeric aluminum (Al_13_O_4_(OH)_24_(H_2_O)_12_^7+^). Metal aluminum not only brings serious pressure to the ecological environment, but also affects human health [34]. From Table 2, it can be seen that the content of metallic aluminum in the grinding fluid increases sharply after barrel finishing. However, the content of metallic aluminum in the grinding fluid decreases dramatically after filtration and separation. And, the content of metallic aluminum of solution A5 is lower than solution A1. This shows that the metal aluminum in barrel finishing can be adsorbed on GO, and the metal aluminum can be taken away from the grinding fluid after separation and filtration. The reason is that GO with specific surface areas and a large number of active functional groups has the advantages of fast equilibrium speed and large adsorption capacity, which can effectively remove heavy metals from aqueous solutions.

Anionic surfactants refer to surfactants with negatively charged structures that have surface active functions. They exist in the form of colloidal particles in aqueous solution. The SDBS as an anionic surfactant is the main component of the grinding fluid, with a content of 1000 mg/L. The anionic surfactants of solutions A1, A5, and A6 are far less than 1000 mg/L, as shown in Table 1. This indicates that GO and SDBS can self-assemble to form new molecules, reducing the content of free anionic surfactants in the solution. It also shows that the reuse process of grinding fluid does not cause the accumulation of anionic surfactants.

Total dissolved solids is the total amount of various ions, molecules, and compounds dissolved in water, except for suspended solids and dissolved gases. The total dissolved solids content of solution A6 is higher than that of solution A1, but lower than that of solution A5. It shows that certain inorganic and organic substances are generated during barrel finishing, and GO with good adsorption ability can adsorb approximately 2000 mg/L of soluble substances.

### 3.3. Water Quality Evaluation of Reused Grinding Fluid

In order to analyze deeply the feasibility of reusing grinding fluids, the chemical indicators (such as COD, pH, total hardness, metal aluminum, anionic surfactants, and total dissolved solids) were set as water quality indicators. Referring to the evaluation method for drinking water quality, each water quality evaluation indicator was assigned a weight (*w_i_*) [35]. Then, the relative weight (*W_i_*) was calculated based on this weight. The estimated quality level based on the drinking water standards of the WHO (2011), GSA (2013), and GB5749-2006 [36] is then determined. The values of parameters that have a significant impact on water quality are designated as the highest value of five, while those with less impact are designated as the lowest value of one. The *W_i_* is calculated using Formula (1) as follows:

(1) $$W_{i}=\frac{w_{i}}{\sum_{i=1}^{n}w_{i}}$$

In the equation, *n* is the parameter quantity. The value *q_i_* is calculated using Formula (2) as follows:

(2) $$q_{i}=\frac{C_{i}}{S_{i}}\times 100$$

In the formula, *C_i_* is the water quality parameter, and *S_i_* is the standard value of the parameter. Finally, the water quality index (*WQI*) is obtained through Formula (3).

(3) $$WQI=\sum_{i=1}^{n}q_{i}\times W_{i}$$

From these, the water quality parameters are shown in Table 3.

**Figure 6:**
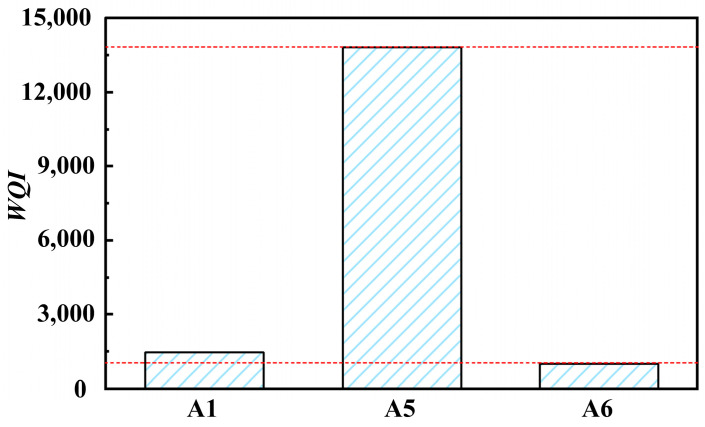
Water quality index of solutions (A1, A5, A6).

The WQI values of solutions A1, A5, and A6 were obtained, as shown in Figure 6. The water quality index value greatly increases after barrel finishing. However, the water quality index value rapidly decreases after physical separation, with a decrease rate of about 90%. Through simple wastewater treatment, grinding fluid cannot meet the requirements of direct discharge of wastewater. However, it can still effectively control water quality and ensure the feasibility of the reuse of grinding fluid.

### 3.4. Reusing Grinding Fluid on Finishing Effects

**Figure 7:**
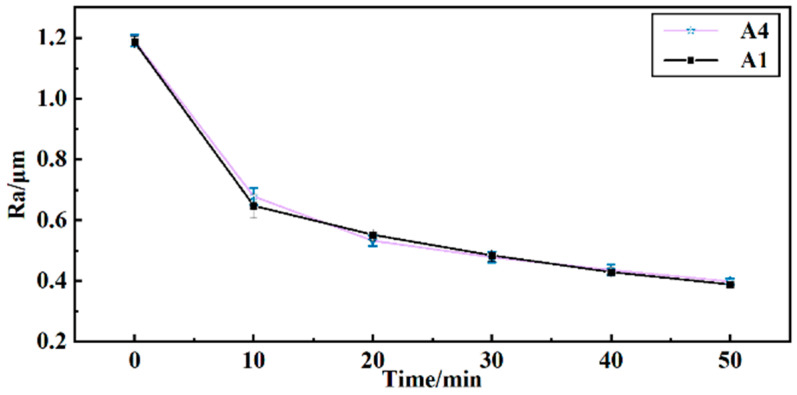
Curves of surface roughness *Ra* of workpiece with time.

To determine the finishing effectiveness of reusing grinding fluid, barrel finishing experiments were conducted using the surface roughness *Ra* of the workpiece as an indicator. As shown in Figure 7, the surface roughness *Ra* was obtained after 50 min of finishing. As per the effect of solution A4, the surface roughness of the workpiece changes from 1.191 μm to 0.398 μm. Per the effect of solution A1, the surface roughness of the workpiece changes from 1.186 μm to 0.388 μm. The surface roughness curve of A4 is similar to the surface roughness curve of A1. This indicates that the reused grinding fluid can still effectively finish the workpiece. The reused grinding fluid also ensures minimal contact force between the workpiece and media during the barrel finishing process. A better surface roughness was obtained with minimal contact force in barrel finishing [1].

## 4. Conclusions

Based on the high COD values of grinding fluids in finishing, the impact of grinding fluid components on COD was analyzed. The water quality when reusing grinding fluid was evaluated by characterization and the finishing effectiveness of grinding fluid with GO was verified by experimentation. The results are summarized as follows:

1. Colloidal SDBS being the surfactant is the main factor affecting the COD values of grinding fluid. GO can adsorb a certain amount of SDBS and take it away. Aiming to decrease the COD values, the use of surfactants should be reduced in grinding fluid for finishing.
2. The decrease rate of WQI after physical separation of grinding fluid is about 90%. GO with adsorption properties and can take impurities (such as metal aluminum and anionic surfactants) away from the grinding fluid. Water quality when reusing grinding fluid can be guaranteed.
3. As per the effect of the reused grinding fluid (solution A4), the surface roughness Ra of the workpiece is as low as 0.398 μm, which is similar to the effect of grinding fluid (solution A1). It indicates that the self-assembly property of molecules in grinding fluid shows stability and the reused grinding fluid has finishing effectiveness.

## Figures and Tables

**Table 1:** The specific information of liquid medium.

Solution	Composition	Barrel Finishing	Physical Separation Filtration
A1 (grinding fluid of finishing)	Deionized water (400 mL), GO (0.2 g), SDBS (0.4 g)	— —	—
A2	Solution A1 (400 mL)	Finishing 50 min	— —
A3 (reusing water resources)	— —	— —	By physical separation filtration solution A2
A4 (reusing grinding fluid of finishing)	Solution A3 (400 mL), GO (0.2 g), SDBS (0.4 g)	— —	— —
A5	Solution A4 (400 mL)	Finishing 50 min	
A6 (reusing water resources)	— —	— —	By physical separation filtration solution A5
B1 (GO solution)	Deionized water (400 mL), GO (0.2 g)	— —	— —
B2 (GO solution)	Deionized water (400 mL), GO (0.2 g)	Finishing 50 min	— —
C1 (SDBS solution)	Deionized water (400 mL), SDBS (0.4 g)	— —	— —
C2 (SDBS solution)	Deionized water (400 mL), SDBS (0.4 g)	Finishing 50 min	— —

**Table 2:** Water quality indicators of solutions (A1, A5, A6).

Water Quality Indicators	A1	A5	A6
pH	7.34	7.09	7.94
Total hardness (mg/L)	84	126	168
Metal aluminum (mg/L)	8.51	193.90	6.78
Anionic surfactants (mg/L)	15.80	6.59	6.14
Total dissolved solids (mg/L)	2000	4400	2400

**Table 3:** Water quality parameters.

*C_i_*	*S_i_* (mg/L)	*w_i_*	*W_i_*
COD	100	5	0.227
pH	8.5	4	0.182
Total hardness	450	2	0.091
Metal aluminum	0.2	3	0.136
Anionic surfactants	0.3	3	0.136
Total dissolved solids	1000	5	0.227

## Data Availability

The raw data supporting the conclusions of this article will be made available by the authors on request.
